# Global analysis of primary mesenchyme cell cis-regulatory modules by chromatin accessibility profiling

**DOI:** 10.1186/s12864-018-4542-z

**Published:** 2018-03-20

**Authors:** Tanvi Shashikant, Jian Ming Khor, Charles A. Ettensohn

**Affiliations:** Department of Biological Sciences, Carnegie Mellon University, Pittsburgh, Pennsylvania USA

**Keywords:** Primary mesenchyme cells, Sea urchins, Skeletogenesis, Gene regulatory network, DNase-seq, ATAC-seq

## Abstract

**Background:**

The developmental gene regulatory network (GRN) that underlies skeletogenesis in sea urchins and other echinoderms is a paradigm of GRN structure, function, and evolution. This transcriptional network is deployed selectively in skeleton-forming primary mesenchyme cells (PMCs) of the early embryo. To advance our understanding of this model developmental GRN, we used genome-wide chromatin accessibility profiling to identify and characterize PMC *cis*-regulatory modules (CRMs).

**Results:**

ATAC-seq (Assay for Transposase-Accessible Chromatin using sequencing) analysis of purified PMCs provided a global picture of chromatin accessibility in these cells. We used both ATAC-seq and DNase-seq (DNase I hypersensitive site sequencing) to identify > 3000 sites that exhibited increased accessibility in PMCs relative to other embryonic cell lineages, and provide both computational and experimental evidence that a large fraction of these sites represent bona fide skeletogenic CRMs. Putative PMC CRMs were preferentially located near genes differentially expressed by PMCs and consensus binding sites for two key transcription factors in the PMC GRN, Alx1 and Ets1, were enriched in these CRMs. Moreover, a high proportion of candidate CRMs drove reporter gene expression specifically in PMCs in transgenic embryos. Surprisingly, we found that PMC CRMs were partially open in other embryonic lineages and exhibited hyperaccessibility as early as the 128-cell stage.

**Conclusions:**

Our work provides a comprehensive picture of chromatin accessibility in an early embryonic cell lineage. By identifying thousands of candidate PMC CRMs, we significantly enhance the utility of the sea urchin skeletogenic network as a general model of GRN architecture and evolution. Our work also shows that differential chromatin accessibility, which has been used for the high-throughput identification of enhancers in differentiated cell types, is a powerful approach for the identification of CRMs in early embryonic cells. Lastly, we conclude that in the sea urchin embryo, CRMs that control the cell type-specific expression of effector genes are hyperaccessible several hours in advance of gene activation.

**Electronic supplementary material:**

The online version of this article (10.1186/s12864-018-4542-z) contains supplementary material, which is available to authorized users.

## Background

A central challenge of developmental biology is to elucidate the relationship between genotype and phenotype. The genomically encoded instructions for development can be represented as gene regulatory networks (GRNs), which consist of combinatorial interactions among regulatory genes (i.e., genes that encode transcription factors) and which specify cell identities through the programs of differential gene expression that they control [1, 2]. GRNs are proving to be powerful tools for understanding the genetic regulation and evolution of development. Establishing a connection between genes and anatomy, however, will require linking transcriptional programs to the cell behaviors that drive specific morphogenetic processes.

The development of the sea urchin endoskeleton is a valuable model for dissecting the genetic control of a morphogenetic process [3–5]. The skeleton is a biomineral composed of calcite and occluded matrix proteins and is secreted by primary mesenchyme cells (PMCs), descendants of the micromeres of the 16-cell stage embryo. During gastrulation, PMCs undergo a sequence of well-described behaviors that includes epithelial-mesenchymal transition, directional migration, cell-cell fusion, and biomineral formation [6–8]. The skeleton influences the shape, orientation, swimming, and feeding of the larva [9, 10].

The GRN that underlies PMC specification is one of the most comprehensive in any developing animal [4, 11]. The PMC GRN is activated by maternal factors localized at the vegetal pole of the egg [12, 13]. These maternal factors act through a double-repression system involving the transcriptional repressor, *pmar1/micro1* [14, 15] to activate a small set of early zygotic regulatory genes, including *ets1* [16] and *alx1* [17, 18], selectively in the large micromere-PMC lineage. The products of these genes engage additional layers of regulatory genes and interactions among the various regulatory genes stabilize the transcriptional network and drive it forward [11]. Recently, high-throughput methods have been used to identify hundreds of downstream effector genes in the PMC GRN [19–21]. These effectors regulate critically important aspects of skeletal morphogenesis, including PMC guidance, PMC fusion, and biomineral formation [22–28]. The sea urchin skeletogenic GRN is understood in sufficient detail that it is currently being leveraged to explore the evolution of skeletogenesis within echinoderms, which exhibit diverse patterns of skeletogenesis [4, 29, 30].

Currently, the developmental GRNs that have been constructed for sea urchins, including the PMC GRN, comprise positive and negative regulatory interactions that have been revealed by perturbing the functions of specific regulatory genes and measuring effects on the expression of other genes in the network. Thus, they are maps of functional (epistatic) interactions that, in most cases, do not discriminate between direct and indirect interactions. For a relatively small number of genes, detailed mutational studies of CRMs have been carried out using reporter constructs and direct transcriptional inputs have been identified. For example, with respect to the PMC GRN, CRMs of *Sp-sm50* [31], *Sp-sm30a* [32, 33], *Sp-tbr* [34], and *Sp-alx1* [35] have been experimentally dissected to varying degrees.

A roadblock to a more detailed understanding of the architecture of this (and other) developmental GRNs is the challenge of identifying relevant CRMs. Experimental analysis of CRMs is currently the gold-standard for elucidating direct interactions between specific regulators and their target genes [1]. Therefore, the high-throughput identification of CRMs is a critically important step in developing more comprehensive GRN models. Evolutionary conservation has been used to assist in the identification of putative CRMs [36], but by itself this approach is less than satisfactory. Methods have also been developed that allow multiplexing of barcoded reporters to facilitate cis-regulatory analysis [37, 38], but these are technically challenging and would benefit from accurate, high-throughput methods for CRM identification.

Genome-wide techniques such as ATAC-seq and DNase-seq have been used to identify regions of open chromatin in a variety of cell types [39–41]. These methods rely on the local depletion of nucleosomes at promoters and CRMs and the resultant hypersensitivity of these regions  to enzymes such as DNase I and Tn5 transposase. Hypersensitive DNA fragments are selectively isolated, sequenced, and mapped to the genome. Several studies have shown that cultured cell lines and adult tissues have patterns of chromatin accessibility characteristic of those cell types [42–45]. In addition, cell type-specific patterns of chromatin accessibility have been used as a primary criterion for CRM discovery [46–48].

In order to obtain a more complete understanding of the gene regulatory program deployed in the PMC lineage, we set out to identify relevant functional CRMs in a high-throughput manner and reveal potential regulatory inputs. We used a combination of ATAC-seq and DNase-seq to identify CRMs that regulate gene expression in the skeletogenic lineage and showed that a large fraction of these CRMs drive PMC-specific expression of GFP reporter plasmids. Our work identifying CRMs selectively active in PMCs will facilitate a comprehensive dissection of this important model developmental GRN and an improved understanding of GRN architecture more broadly. Our studies also reveal a surprising developmental history of a large suite of lineage-specific CRMs, which we find are hyperaccessible several hours before cell type-specific transcripts are expressed and are open in multiple embryonic cell lineages. The latter may reflect the pluripotency of early sea urchin embryonic cells or the association of these CRMs with repressors in non-skeletogenic cells.

## Results

### Global analysis of hyperaccessible sites in PMCs using ATAC-seq

**Fig. 1:**
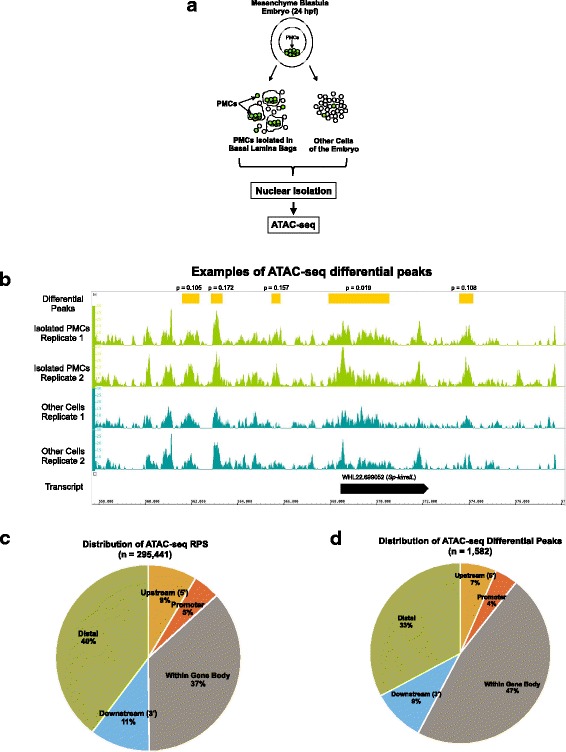
ATAC-seq sample preparation and sequence analysis. **a** *S. purpuratus* embryos were cultured for 24 h at 15 °C in triplicate. PMCs and other cells were isolated and ATAC-seq libraries were generated and sequenced. Sequence reads were analyzed by the bioinformatics pipeline shown in Additional file 1: Fig. S1A. **b** Examples of ATAC-seq differential peaks. These differential peaks (yellow rectangles) are located near the *Sp-kirrelL* gene, which encodes a transmembrane protein required for PMC fusion [27]. The aligned reads for each replicate are visualized, and the difference in peak magnitudes can be seen when comparing differential peaks in the isolated PMC replicates (light green peak trace) to the other cell replicates (dark green trace). Nominal *p*-values for differential peaks are indicated. **c** The distribution of ATAC-seq peaks in the RPS with respect to the closest gene. See Methods for definitions of peak locations. **d** Distribution of ATAC-seq differential peaks with respect to the closest gene

PMCs and a “non-PMC” cell fraction were isolated from early mesenchyme blastula stage embryos at 24 h post-fertilization (hpf) as described previously [21]. As in this previous study, the purity of the PMC fraction was > 90% as determined by the fraction of 6a9-positive cells, and the depletion of PMCs from the non-PMC fraction was confirmed by RT-PCR. ATAC-seq was performed on three biological replicates from separate matings. Each replicate consisted of two samples: isolated PMCs and all other (non-PMC) cells (Fig. 1a). A total of six Illumina libraries were generated and sequenced. Sequence reads were analyzed using a bioinformatics pipeline consisting of open-source tools and two custom Python programs (Additional file 1: Figure S1A; see Methods for analysis details and Additional file 2: Table S1 for additional information on sequencing). On average, 89 million 76 bp single-end reads were obtained per sample, of which 69 million reads (77.5%) mapped to the *S. purpuratus* genome (Table 1). Two sets of replicates were highly concordant, with an average pairwise Pearson’s correlation coefficient of 0.915 (Additional file 1: Figure S1B). One replicate, however, was less concordant (Pearson’s correlation coefficient threshold ≤0.8) and was therefore not included in the analysis. A reference peak set (RPS) was generated from the two highly concordant replicates by identifying replicate peaks that overlapped by at least 75% non-reciprocally and then merging all such peaks across the two samples (see Methods and Additional file 3: Table S2). This RPS contained 295,441 peaks (average peak size ~ 600 bp) that covered 18.84% of the genome. The PMC RPS, provided in Additional file 4: Table S3, represents a comprehensive map of hyperaccessible sites in PMCs at the mesenchyme blastula stage, when the great majority of the genes in the PMC GRN are expressed [21]. A small fraction (5%) of these hyperaccessible sites marked gene promoters but most peaks were associated with intergenic regions or introns and represented putative CRMs (Fig. 1c and see below). Previous RNA-seq data have shown that PMCs express ~ 9500 genes at this developmental stage (FPKM greater than or equal to 5.0) (Rafiq et al., 2014); thus, there are approximately 30 hyperaccessible sites/expressed gene. A RPS was also generated for the Other Cell (non-PMC) cell fraction (Additional file 5: Table S4).

**Table 1:** Sequence Analysis Details for ATAC-seq Samples

Avg. no. sequence reads/sample	89 M
Avg. no. reads mapped/sample	69 M (77.5%)
Avg. no. reads/sample after duplicate removal and equalization	43 M (48.3%)
Avg. no. peaks called/sample	367,113
Avg. fraction reads in peaks	0.635
No. peaks in RPS	295,441
Avg. size of peak in RPS	597 bp
Genome coverage of RPS	18.84%
No. differential peaks (*p* < 0.2)	1582
No. differential peaks within 10 kb of genes	1063
No. differential peaks within 10 kb of PMC DE genes	275; 5.99-fold enrichment (*p* < 2.2e-16)

One thousand five hundred eighty two peaks with nominal *p*-values < 0.2 as calculated by DESeq2 [85] were determined to be enriched in the isolated PMCs compared to the other non-PMC cells: these are ATAC-seq differential peaks. Two hundred seventy five of the 1582 differential peaks were found to be within 10 kb of PMC DE genes, a highly significant enrichment

Differential chromatin accessibility has been shown to be a useful criterion for the discovery of cell type-specific enhancers [46–48]. We therefore analyzed our ATAC-seq data to identify regions of chromatin that were differentially hyperaccessible in PMCs (see Methods). One thousand five hundred eighty two peaks were identified that had significantly elevated signal in isolated PMCs compared with the non-PMC (“Other Cell”) cell fraction. We refer to these peaks as “ATAC-seq differential peaks” (see Fig. 1b for examples and Additional file 6: Table S5 for the coordinates of all ATAC-seq differential peaks identified). As there are approximately 400 genes differentially expressed by PMCs at this stage (“PMC DE genes”) [21], this represents about 4 ATAC-seq differential peaks/PMC DE gene. Of the 1582 ATAC-seq differential peaks, we found that 275 peaks were within 10 kb of PMC DE genes. Differential peaks were much more likely to be located within 10 kb of DE genes than non-differential peaks; this difference was significant by Fisher’s exact test (*p*-value < 2.2e-16; 5.99-fold enrichment). This strongly suggested that cell type-specific accessibility of peaks was a useful criterion for the identification of CRMs that control genes differentially expressed by PMCs. We also identified ~1,500 peaks that were differentially accessible in the non-PMC cell fraction (Additional file 7: Table S19).

Of the 1582 ATAC-seq differential peaks, 1063 were within 10 kb of at least one gene. Of the 1110 genes in this set, 326 have been assigned to functional (GO) categories, as annotated in Echinobase [49–51]. Biomineralization and metalloprotease functional categories were highly enriched in genes that were within 10 kb of ATAC-seq differential peaks, as compared with the set of genes that were within 10 kb of non-differential peaks (adjusted Fisher’s exact test *p*-value < 0.05; 3.26-fold avg. enrichment) (see Additional file 8: Figure S2A). Biomineralization is the principal biological function of PMCs and biomineralization genes constitute the largest class of PMC DE genes [21]. In addition, pharmacological studies have shown that metalloprotreases play a critically important role in skeletogenesis [52, 53]. Thus, the enrichment of differential peaks near biomineralization and metalloprotease genes was consistent with their enrichment near PMC DE genes more generally and supported the view that cell type-specific chromatin accessibility was a valuable indicator of CRMs active in PMCs.

The genes closest to the peaks in the RPS were identified and the location of each peak with respect to the closest gene was determined (see Methods). Of all peaks in the ATAC-seq RPS, 40% were distal, 37% were within gene bodies, 11% were downstream of genes, 9% were upstream of genes and 5% were closely associated with putative promoter regions (Fig. 1c). Of the 1582 ATAC-seq differential peaks, 33% were distal, 47% were within gene bodies, 9% were downstream of genes, 7% were upstream of genes and 4% were closely associated with putative promoter regions (Fig. 1d). This represented an enrichment of ATAC-seq differential peaks within gene bodies (Fisher’s exact *p*-value = 1.10e-15; 1.29-fold enrichment) and a depletion in distal regions (Fisher’s exact p-value = 9.25e-08; 1.21-fold depletion) compared to the RPS.

### Analysis of U0126-dependent, hyperaccessible sites identified by DNase-seq

As a parallel, independent approach to the identification of regions of chromatin differentially accessible in PMCs, we used DNase-seq in combination with pharmacological ablation of PMCs. We used U0126, a well-characterized MEK inhibitor, which selectively blocks PMC specification [54, 55]. Whole-embryo RNA-seq analysis has confirmed that U0126 selectively inhibits the expression of genes differentially expressed by PMCs when the inhibitor is used at early developmental stages [21], although we cannot rule out the possibility that this drug has  limited effects on other tissues. Our rationale for applying this secondary approach was our expectation that putative PMC CRMs that were identified by two completely independent methods would constitute a high confidence subset that would be useful for further computational and experimental analyses, as described below.

**Fig. 2:**
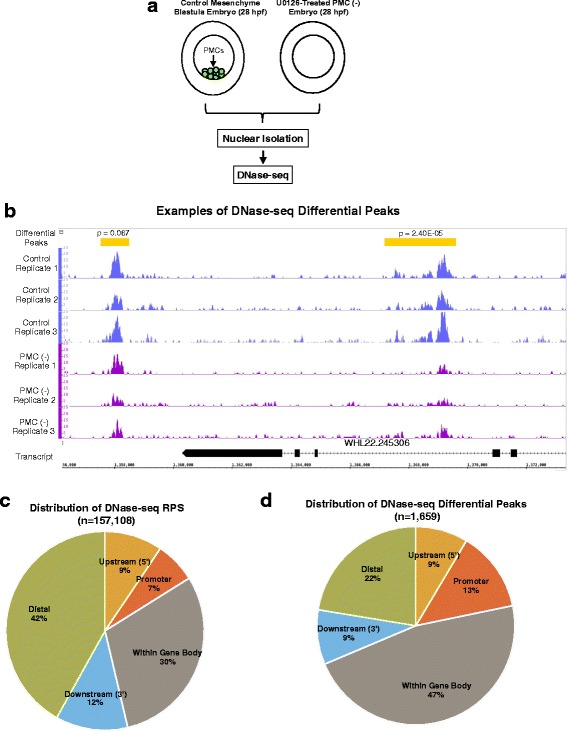
DNase-seq sample preparation and sequence analysis. **a** *S. purpuratus* embryos were treated with U0126 at the 2-cell stage to obtain PMC (−) embryos. Control and U0126-treated embryos were cultured for 28 h at 15 °C in triplicate. Nuclei were isolated and DNase-seq was carried out. Sequence reads were analyzed by the bioinformatics pipeline shown in Additional file 1: Figure S1A**. b** An example of DNase-seq differential peaks. The differential peaks (yellow rectangles) are located near the WHL22.245306 transcript. The aligned reads for each replicate are visualized as traces, and the differences in peak magnitude are clear when comparing control whole embryos (violet peak trace) to PMC(−) embryos (dark purple trace). Nominal p-values for differential peaks are indicated. **c** Distribution of DNase-seq peaks in the RPS with respect to the closest gene. See Methods for definitions of peak locations. **d** Distribution of DNase-seq differential peaks with respect to the closest gene

DNase-seq was performed on three biological replicates from separate matings. Each replicate consisted of two samples: control mesenchyme blastulae and sibling U0126-treated, PMC (−) embryos (Fig. 2a). A total of six Illumina libraries were generated and sequenced. We found that peaks identified in replicate samples were highly concordant, with an average pairwise Pearson’s correlation coefficient of 0.955 (Additional file 1: Figure S1C). On average, 23.5 million 50 bp single-end reads were obtained per sample, of which 19 million reads (80.8%) were mapped to the *S. purpuratus* genome (Table 2 and see Additional file 9: Table S6 for additional information on sequencing). One thousand six hundred fifty nine peaks were identified that had significantly elevated signal (nominal *p*-value < 0.1) in control embryos compared to PMC (−) embryos. We refer to such peaks as “DNase-seq differential peaks” (see Fig. 2b for an example and Additional file 10: Table S7 for coordinates of all DNase-seq differential peaks identified). We also identified 817 peaks that were differentially accessible in PMC (-) embryos relative to control embryos (Additional file 11: Table S20 and Additional file 12: Table S21).

**Table 2:** Sequence Analysis Details for DNase-seq Samples

Avg. no. sequenced reads/sample	23.5 M
Avg. no. reads mapped/sample	19 M (80.8%)
Avg. no. reads mapped/sample after duplicate removal and equalization	14.7 M (62.5%)
Avg. no. peaks called/sample	256,007
Avg. fraction reads in peaks	0.55
No. peaks in RPS	157,108
Avg. size of peak in RPS	637 bp
Genome coverage of RPS	10.68%
No. differential peaks (*p* < 0.1)	1659
No. differential peaks within 10 kb of a gene	1287
No. differential peaks within 10 kb of a PMC DE gene	258; 5.31-fold enrichment (*p* < 2.2e-16)

One thousand six hundred fifty nine peaks with nominal p-values < 0.1 as calculated by DESeq2 [85] were determined to be enriched in the control embryos compared to PMC (−) embryos: these are DNase-seq differential peaks. Two hundred fifty eighty of these peaks were found to be within 10 kb of PMC DE genes

As we found with differential ATAC-seq peaks, differential DNase-seq peaks were much more likely to be located near PMC DE genes (and biomineralization genes) than non-differential peaks. Of the 1659 DNase-seq differential peaks, 1287 peaks were within 10 kb of at least one gene. Of these DNase-seq differential peaks, 20% (258/1287) were within 10 kb of PMC DE genes, a significant enrichment compared with non-differential peaks (Fisher’s exact test, *p*-value < 2.2e-16; 5.31-fold enrichment). Of the 1216 genes within 10 kb of DNase-seq differential peaks, 400 have been assigned to functional (GO) categories, as annotated in Echinobase [49–51]. Biomineralization and transcription factor functional categories were highly enriched in this gene set compared to genes located near non-differential peaks (adjusted Fisher’s p-value <8e-07, 3.46-fold avg. enrichment) (see Additional file 8: Figure S2B). The finding that DNase-seq differential peaks were much more likely than non-differential peaks to be located near DE (and biomineralization) genes provided evidence that U0126 sensitivity was a useful indicator of CRMs active in PMCs.

We mapped the location of each peak in the RPS relative to the nearest gene (see Methods). We found that 42% of RPS peaks were distal, 30% were within gene bodies, 12% were downstream of genes, 9% were upstream of genes and 7% were closely associated with putative promoter regions (Fig. 2c). Of the 1659 DNase-seq differential peaks, 22% were distal, 47% were within gene bodies, 9% were downstream of genes, 9% were upstream of genes and 13% were closely associated with putative promoter regions (Fig. 2d). Of the peaks found within the gene body (both in the RPS and in the set of DNase-seq differential peaks), the majority (∼90%) were in introns. These data revealed a significant enrichment of DNase-seq differential peaks in promoter regions (Fisher’s exact *p*-value = 1.10e-15; 1.97-fold enrichment) and within gene bodies (Fisher’s exact p-value = 1.10e-15; 1.55-fold enrichment) and a significant depletion of distal peaks (Fisher’s exact *p*-value = 1.10e-15; 1.32-fold depletion) relative to the RPS. The latter two findings mirrored our observations with differential ATAC-seq peaks (above).

We examined the extent to which the data obtained by the two independent chromatin accessibility mapping methods (ATAC-seq and DNase-seq) were congruent (Table 3). Overall, when we compared the RPSs derived from DNase-seq and ATAC-seq data, we observed a high degree of correspondence. A very large fraction of the DNase-seq peaks (88%) overlapped ATAC-seq peaks by at least 1 nt. We noted that the total number of peaks in the ATAC-seq RPS was almost twice that of the DNase-seq RPS (295,441 and 157,108 peaks, respectively), although the average peak sizes of the two RPSs were very similar (597 and 637 bp, respectively). The larger number of called peaks in the ATAC-seq RPS may have been due to the greater depth of sequencing and/or to a slightly lower level of noise in these data (we found that the average FRIP score was slightly higher in the ATAC-seq data).

**Table 3:** Correspondence Between ATAC-seq and DNase-seq Datasets

No. peaks in DNase-seq RPS	157,108
No. peaks in ATAC-seq RPS	295,441
No. of DNase-seq RPS peaks overlapping ATAC-seq RPS peaks	138,145 (88% of DNase-seq RPS)
No. of ATAC-seq RPS peaks overlapping DNase-seq RPS peaks	130,552 (44% of ATAC-seq RPS)
No. of DNase-seq differential peaks	1659 peaks
No. of ATAC-seq differential peaks	1582 peaks
No. of overlapping differential peaks	161 (*p* = 2.2e-16)
No. of overlapping differential peaks within 10 kb of PMC DE genes	73 (*p* < 5.5e-10; 2.38-fold avg. enrichment)

One hundred sixty one peaks are present in both the DNase-seq and ATAC-seq differential peak set, overlapping by at least 75% in one direction. Of these 161 overlapping peaks, 73 are within 10 kb of PMC DE genes

### Differential chromatin accessibility mapping identifies known PMC CRMs

**Fig. 3:**
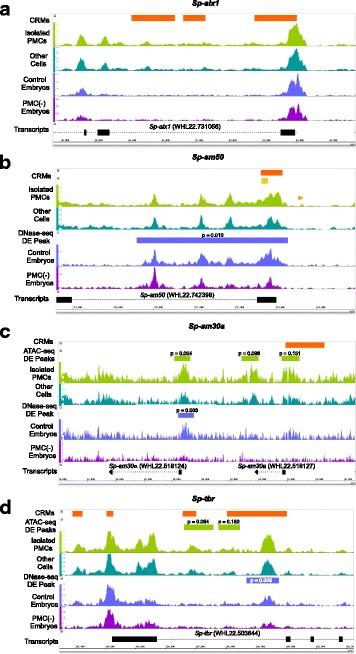
Previously analyzed PMC-specific *cis*-regulatory modules. Previously analyzed *cis*-regulatory modules (CRMs) (orange and yellow rectangles) that control the spatio-temporal expression of four PMC DE genes are represented in both ATAC-seq (light green trace: isolated PMCs; dark green trace: other cells) and DNase-seq (violet trace: control whole embryos, purple trace: U0126-treated embryos) datasets. **a** *Sp-alx1 cis*-regulatory modules are not represented as differential peaks in the ATAC-seq or DNase-seq datasets. **b** The *Sp-sm50* enhancer (orange rectangle) and the minimal element (yellow rectangle) required for correct spatio-temporal expression of *Sp-sm50* are encompassed within a differential peak identified in the DNase-seq dataset (violet rectangle), but not identified as differential in the ATAC-seq dataset. **c** The *Sp-sm30a* enhancer overlaps a differential peak identified in the ATAC- seq dataset (light green), but is not identified as differential in the DNase-seq dataset. **d** Two of four previously studied *Sp-tbr cis*-regulatory modules overlap 2 differential peaks in the ATAC-seq (light green) dataset and 1 differential peak in the DNase-seq (violet) dataset

CRMs that regulate four genes expressed selectively by PMCs have been identified by low-throughput approaches and experimentally verified through mutational analysis of reporter constructs. The genes are: *Sp-sm50* [31], *Sp-sm30a* [32, 33], *Sp-tbr* [34], and *Sp-alx1* [35]. Each of these experimentally verified CRMs aligned with local regions of open chromatin in one or both of the ATAC-seq and DNase-seq datasets (Fig. 3). The *Sp-alx1* CRMs (Fig. 3a) were not identified as significantly differentially hypersensitive in either dataset. The *Sp-sm50* CRM (Fig. 3b) was differentially hypersensitive in the DNase-seq dataset but not in the ATAC-seq dataset, while the *Sp-sm30a* CRM (Fig. 3c) was differentially hypersensitive in the ATAC-seq dataset but not in the DNase-seq dataset. Of the four known CRMs involved in regulating *Sp-tbr* expression (Fig. 3d), one overlapped an ATAC-seq differential peak and one overlapped both DNase-seq and ATAC-seq differential peaks. These observations showed that known PMC CRMs were well represented in the combined set of differential peaks obtained by DNase-seq and ATAC-seq. They also showed, however, that our identification of PMC CRMs was not exhaustive by either DNase-seq or ATAC-seq and that the most complete capture of known CRMs came from combining approaches.

### Validation of newly discovered PMC CRMs using GFP reporter gene assays

**Fig. 4:**
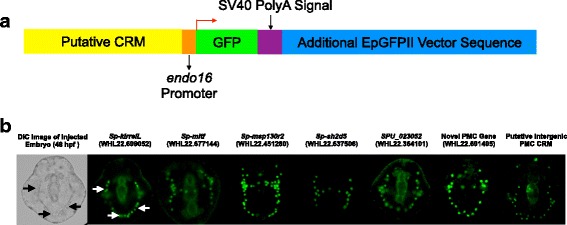
Experimental validation of putative PMC CRMs. **a** The EpGFPII reporter construct: Of a total of 3073 PMC-enriched differential peaks identified using DNase-seq and ATAC-seq, 31 were cloned into the EpGFPII plasmid, upstream of the GFP coding sequence and the *Sp-endo16* promoter, and injected into *S. purpuratus* eggs. **b** Representative images of *S. purpuratus* embryos injected with 7 reporter constructs, showing PMC-specific GFP expression (green fluorescence) at 48 hpf. DIC and *Sp-kirrelL* images show the same embryo; arrows indicate PMCs

To validate our experimental and computational identification of PMC CRMs, 31 candidate CRMs were cloned into the EpGFPII plasmid [56] upstream of the *Sp-endo16* promoter (Fig. 4a). We focused primarily, but not exclusively, on peaks that were differentially accessible in both the ATAC-seq and DNase-seq datasets and that were also within 10 kb of PMC DE genes (see Additional file 13: Table S8 for detailed information on all CRMs tested). Reporter plasmids were injected into fertilized *S. purpuratus* eggs and GFP expression was assayed by fluorescence microscopy at 48 hpf. 9/31 constructs (29%) expressed GFP at detectable levels (Fig. 4b, Table 4). Significantly, all 9 of these reporters drove expression of the reporter gene only in PMCs i.e., none of the constructs we tested resulted in detectable levels of GFP expression in other cell types. The high proportion of active CRMs that showed cell type-specific expression provided a powerful experimental validation of our approach. It should be noted that the reporter assay was a stringent one which required that a putative CRM was, by itself, sufficient to direct robust, spatially correct expression. Many sea urchin genes are controlled by multiple CRMs, some of which function only to modulate the timing or the level of gene expression [34, 35, 57], and such elements would not be expected to be active in our assay.

**Table 4:** PMC CRMs Validated by Reporter Gene Assays

Putative CRM Tested	Total No. Injected Embryos	No. GFP-Positive Embryos	No. Embryos Expressing GFP in PMCs
*Sp-kirrelL* (WHL22.699052)	288	88 (31%)	88
*Sp-mitf* (WHL22.677144)	230	170 (74%)	170
*Sp-msp130r2* (WHL22.451280)	244	106 (43%)	106
*Sp-sh2d5* (WHL22.637506)	80	43 (54%)	43
*SPU_023052* (WHL22.364101)	149	28 (19%)	28
Novel PMC DE Gene (WHL22.691495)	160	109 (68%)	109
Intergenic	78	35 (45%)	35
*Sp-hypp_2386* (WHL22.239326)	86	53 (62%)	53
*Sp-c-lectin/PMC1* (WHL22.411805)	257	27 (11%)	27

Nine out of 31 injected reporter constructs showed PMC-specific GFP expression at 48 hpf. No ectopic expression was observed

Consensus transcription factor binding sequences have been characterized for fourteen sea urchin transcription factors: Ets1, Alx1, Blimp1, Tbr, Tcf1, Gata, Otx, HesC, bZIP, Sox, Myb, Ot1, Gcm and CBF (Additional file 14: Table S9). We used FIMO [58] to scan the PMC CRMs validated by reporter gene assays for known sea urchin transcription factor consensus binding sequences. Consensus sequences for Sox, Tbr, Gcm, bZIP, Otx, Myb, Ets1, HesC, Blimp1, Gata and Alx1 were identified (Additional file 15: Table S10). These candidate regulators, which include several (Alx1, Ets1 and Tbr) that are known to function in the PMC GRN, can be tested experimentally by mutating the relevant binding sites in the CRMs.

### Computational analysis of high-confidence PMC CRMs

**Fig. 5:**
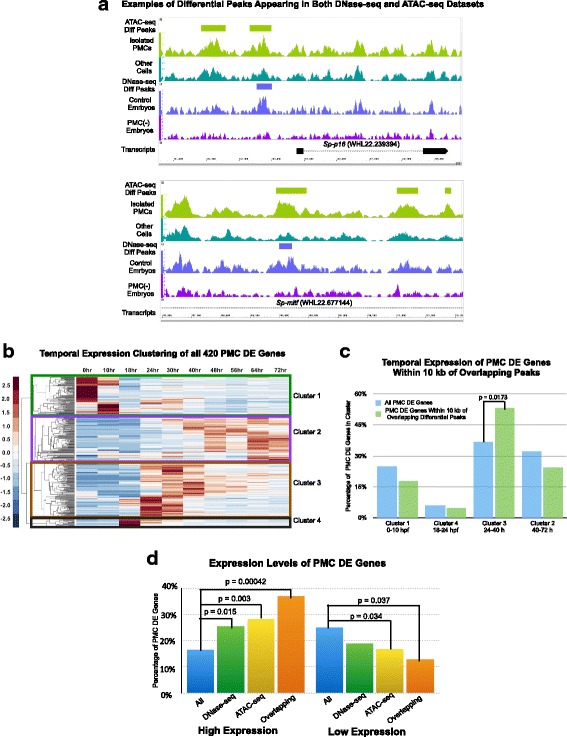
Computational analysis of high-confidence PMC CRMs. **a** ATAC-seq differential peaks (green rectangles) and DNase-seq differential peaks (violet rectangles) near *Sp-p16* and *Sp-mitf*, both PMC DE genes. Aligned reads averaged across replicates, from isolated PMCs (light green trace) and non-PMC cells (dark green trace) using ATAC-seq, and control 28 hpf embryos (violet trace) and PMC (−) embryos (dark purple trace) using DNase-seq, are shown. **b** Temporal expression profiles (Tu et al., 2012) of 420 PMC DE genes identified previously (Rafiq et al., 2014). Each gene is represented by a single row. The color scale ranges from deep red (2.5-fold higher than mean expression) to deep blue (2.5-fold lower than mean expression). White indicates mean expression. Four clusters are delineated, corresponding to maximal gene expression at 0–10, 40–72, 24–40 and 18–24 hpf, respectively. **c** Temporal expression of the 62 PMC DE genes within 10 kb of overlapping differential peaks: these PMC DE genes were classified into four clusters, delineated in Fig. 3b. **d** PMC DE genes were classified into categories based on levels of gene expression in isolated PMCs (data obtained from (Rafiq et al., 2014). “High” expression genes: FPKM between 2512 and 100 (top 17% of all 420 DE genes); “very low” expression genes: FPKM between 14 and 0 (bottom 25% of all 420 DE genes)

To obtain a high-confidence set of PMC CRMs for additional computational analysis, all DNase-seq differential peaks with 75% or more of their sequence overlapping one or more ATAC-seq differential peaks were merged with all ATAC-seq differential peaks that had 75% or more of their sequence overlapping one or more DNase-seq differential peaks. This generated a new set of 161 peaks common to the DNase-seq and ATAC-seq datasets: we call these peaks “overlapping differential peaks” (see Fig. 5a for examples and Additional file 16: Table S11 for the coordinates of all 161 merged, overlapping peaks). Although the number of overlapping differential peaks was not large, we chose stringent conditions (75% overlap) in order to ensure that our computational analysis did not include false positives. The probability that the observed degree of overlap between the DNase-seq differential peaks and the ATAC-seq differential peaks occurred by chance was vanishingly small (*p*-value < 2.2e-16 by Fisher’s exact test), demonstrating that the two independent datasets converged on related populations of differential peaks. The fraction of overlapping differential peaks that were within 10 kb of a PMC DE gene (73/161, or 45%) represented a significant enrichment compared to ATAC-seq and DNase-seq differential peaks as a whole (Fisher’s exact test *p*-value < 5.5e-10; 2.38-fold enrichment). Moreover, genes that were within 10 kb of overlapping differential peaks were greatly enriched for biomineralization genes (adjusted Fisher’s exact *p*-value = 2.73e-12; 19.61-fold enrichment) (see Additional file 8: Figure S2C). These findings strongly supported the view that the overlapping differential peaks represented a high confidence set of CRMs that regulate genes differentially expressed by PMCs.

In a previous study [21], the expression patterns of 420 PMC-enriched transcripts were classified into four clusters based on developmental transcriptome data [59]. (Fig. 5b). Cluster 1 consisted of 104 transcripts with maximal expression between 0 and 10 hpf, cluster 2 consisted of 136 transcripts with maximal expression between 40 and 72 hpf, cluster 3 consisted of 155 transcripts with maximal expression between 24 and 40 h hpf, and cluster 4 consisted of 25 transcripts with maximal expression between 18 and 24 hpf. When we assigned the 62 PMC- enriched transcripts located within 10 kb of overlapping differential peaks to the above clusters, we observed a significant enrichment of these transcripts in Cluster 3 (Fisher’s exact test *p*-value = 0.0173) (Fig. 5c). Cluster 3 genes were expressed maximally at a time that corresponded closely to the developmental stage we used for chromatin accessibility profiling and included a disproportionate number of genes with roles in skeletal development. A corresponding reduction in the proportions of transcripts in Clusters 1 and 2 was also observed, but these differences were not statistically significant.

We also binned the 420 PMC DE genes into four classes based on their expression levels in PMCs at 24 hpf, using RNA-seq data [21]. The “high expression” class (70 genes) had expression levels between 2512 and 100 FPKM, the “medium expression” class (117 genes) had expression levels between 99 and 40 FPKM, the “low expression” class (127 genes) had expression levels between 39 and 15 FPKM, and the “very low expression” class (106 genes) had expression levels between 14 and 0 FPKM. The set of PMC DE genes that were within 10 kb of all differential peak sets showed a different distribution of expression levels than PMC DE genes as a whole. Specifically, genes near overlapping differential peaks were more likely to be in the “high expression” class (Fisher’s exact *p*-value = 0.00042) and less likely to be in the “very low expression” class (Fig. 5d). This finding was consistent with our observation that overlapping differential peaks tended to lie near biomineralization genes, most of which are expressed at high levels in PMCs at this stage [21].

We used AME to determine whether known binding sites for sea urchin transcription factors were enriched in PMC CRMs (see Methods). Binding sites for Ets1 and Alx1, two PMC-enriched transcription factors that have direct or indirect inputs into half of the known PMC effector genes in the PMC gene regulatory network [21], were found to be significantly enriched (*p*-value < 0.0134; Fisher’s exact test) in the overlapping differential peak set (and in the ATAC-seq and DNase-seq differential peak sets), providing additional support for the validity of our CRM identification. No enrichment was observed for binding sites of transcription factors that function primarily in other embryonic cell types (e.g., Gcm, Sox and Gata). AME analysis also showed that binding sites for HesC were significantly enriched (*p*-value = 0.00156; Fisher’s exact test) in the DNase-seq differential peak set. Lastly, we used MEME [60, 61] for the de novo discovery of motifs enriched in the overlapping differential peak set compared to non-differential peaks. Repeating CT (or GA) motifs were found to be highly enriched in the overlapping differential peak set. (Additional file 17: Figure S3).

### PMC CRMs are hyperaccessible at early developmental stages

**Fig. 6:**
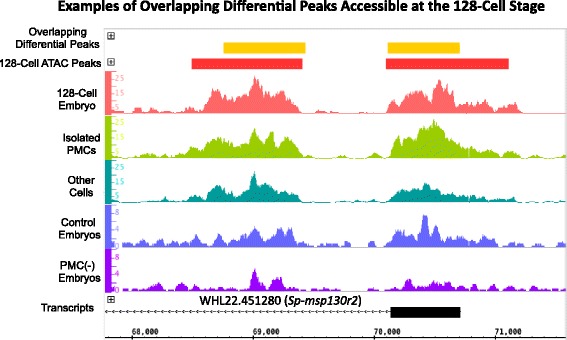
Examples of overlapping differential peaks accessible at the 128-cell stage. Overlapping differential peaks (yellow rectangles) around the *Sp-msp130r* gene are accessible at the 128-cell stage (red rectangles represent peaks called at the 128- cell stage). Hypersensitivity corresponding to the overlapping differential peaks is seen at the 128-cell stage (red trace), the 24 hpf stage isolated PMCs (light green trace) and other non-PMC cells (dark green trace), and control 28 hpf embryos (violet trace) and PMC (−) embryos (dark purple trace)

We performed ATAC-seq on one batch of 128-cell (11 hpf) *S. purpuratus* embryos to investigate whether putative PMC CRMs were accessible during early cleavage, several hours before the majority of skeletogenic lineage genes are expressed (see Additional file 18: Table S12 for additional sequencing information and Additional file 19: Table S13 for coordinates of all peaks accessible at the 128-cell stage). A large number of ATAC-seq and DNase-seq differential peaks were found to be hyperaccessible at the 128-cell stage, including 77/161 (48%) of the overlapping differential peaks (see Fig. 6, Additional file 20: Table S14, and Additional file 21: Table S15). The set of 34 overlapping differential peaks that were not hyperaccessible at the 128-cell stage were similar with respect to their position relative to the closest gene, their proximity to PMC DE genes, and the temporal expression profiles of neighboring PMC DE genes, when compared to the set of overlapping differential peaks as a whole. Of the combined 3073 differential peaks identified using ATAC-seq and DNase-seq, 1267 (41%) were hyperaccessible at the 128-cell stage. Given that the vast majority of PMC DE genes are terminal differentiation genes, it is surprising that 41% of putative PMC regulatory elements are accessible at a stage well before the majority of these genes are expressed.

## Discussion

Our work enhances the value of the PMC GRN as a general paradigm of developmental GRN architecture and evolution, and extends its utility as an experimental model for elucidating the genetic regulation of morphogenesis. Our ATAC-seq-based mapping of PMC chromatin accessibility complements recent RNA-seq-based cataloguing of genes differentially expressed by PMCs [20, 21] and will support a more comprehensive dissection of this network. The high-throughput identification of CRMs associated with skeletogenic effector genes, including a high-confidence set of PMC CRMs identified by two independent approaches, will allow further experimental dissection of direct regulatory inputs into these effectors through mutational analysis of the CRMs. Our studies also confirm that differential chromatin accessibility by itself is a valuable tool for the high-throughput identification of CRMs in early embryonic cells, as has been shown for several terminally differentiated cell types in adult organisms [46, 47].

A variety of evidence supports the conclusion that differential peaks represent CRMs selectively active in PMCs. First, differential peaks in both the ATAC-seq and DNase-seq datasets were much more likely to lie near genes differentially expressed by PMCs than were other peaks (this was also reflected by the increased tendency of differential peaks to lie near biomineralization genes). Our analysis of the overlapping set of differential peaks showed that they were more likely than other PMC DE genes to be expressed at high levels and to exhibit an expression maximum 24–40 hpf, both characteristic features of many genes differentially expressed by PMCs [21]. Indeed, we confirmed that almost half of the functionally annotated genes within 10 kb of overlapping differential peaks were associated with biomineralization, the unique developmental function of PMCs. We identified many specific examples of differential peaks located near well-characterized effectors of skeletal morphogenesis, including *Sp-kirrelL* [27], *Sp-p16* [23], several spicule matrix and MSP130 family genes [62], and carbonic anhydrase [63]. Significantly, consensus binding sites for Ets1 and Alx1, key transcription factors that provide regulatory inputs into almost half of all genes differentially expressed by PMCs [21], were highly enriched in the set of overlapping differential peaks. Most importantly, a large fraction of differential peaks that we tested experimentally (almost 30%) contained sufficient regulatory information to drive reporter gene expression selectively in PMCs, while none supported expression in other cell types. It is important to note that our reporter assay required that a cloned CRM function in isolation to direct cell type-specific expression; this was a stringent test that would not have detected CRMs that act only to modulate the level or timing of gene expression, CRMs that require interactions with other cis-elements in order to function, or insulators. In addition, our visual assay for reporter gene expression was not a highly sensitive one, and we would not have detected weakly active CRMs.

While differential accessibility is a reliable predictor of PMC CRMs, the converse is not true; i.e., absence of differential signal is not strong evidence that a given region of non-coding DNA lacks regulatory function in PMCs. Of course, some CRMs that regulate ubiquitously expressed genes are likely open in all cell types. Even for those genes differentially expressed by PMCs, as discussed below, it seems likely that many of the relevant CRMs are hypersensitive in non-PMC lineages and this may have reduced our ability to detect such regions. We also carried out our analyses at a single developmental stage (28 hpf), and some PMC CRMs might exhibit maximal differential accessibility earlier or later in embryogenesis.

Of the set of previously verified PMC CRMs that regulate *Sp-alx1, Sp-tbr, Sp-sm30a,* and *Sp-sm50*, most were identified as differentially open in our analysis. In several cases, however, these CRMs were detected in either the ATAC-seq dataset or the DNase-seq dataset, but not in both. This reinforces the view that a requirement for differential accessibility in both datasets is a very stringent one and points to the reliability of this subset of peaks. At the same time, it indicates that many additional PMC CRMs were identified as differentially accessible by only one of the two approaches. Indeed, 11 CRMs of this type were tested using reporter gene assays and 3 drove GFP expression specifically in PMCs. In addition, when we considered the 3073 peaks identified as differential by either the ATAC-seq or DNase-seq analysis, nearly 15% of the genes within 10 kb of these peaks were PMC DE genes – a significant enrichment (Fisher’s exact test *p*-value < 2.2e-16; 7.6-fold enrichment). Taken as whole, these considerations suggest that most bona fide skeletogenic GRNs are probably contained in the union of the two individual datasets.

Our findings revealed that, for the most part, the chromatin landscape of PMCs is not highly specific to this cell type. Although we identified reproducible differences in local chromatin accessibility that were predictive of functional CRMs, we rarely observed dramatic differences in peak signals. For example, when we compared the ATAC-seq profiles of purified PMCs and non-PMCs, we consistently observed relatively subtle differences in chromatin accessibility even at CRMs that were subsequently validated experimentally by reporter gene analysis. This strongly suggests that the CRMs of genes expressed specifically by PMCs are open in other cell lineages during early development. It is important to note that we used the same PMC purification method for previous RNA-seq studies and found that FPKM values for PMC-specific mRNAs were typically more than an order of magnitude higher in the PMC fraction than in other cells, confirming the effectiveness of the PMC isolation procedure [21].

One explanation for the hypersensitive state of skeletogenic CRMs in non-PMC lineages may lie in the well-known developmental plasticity of sea urchin embryonic cells. Cell types other than PMCs, including endoderm and non-skeletogenic mesoderm cells, have the capacity to adopt a skeletogenic fate under certain experimental conditions, even late in gastrulation [64–66]. The skeletogenic potential of these cells may be associated with the priming of PMC CRMs. Surprisingly, our findings strongly suggest that this holds true even of CRMs that regulate terminal skeletogenic differentiation genes. Studies on pluripotent embryonic stem (ES) cells have identified primed (poised) enhancers that are characterized by open chromatin and other epigenetic marks, yet are transcriptionally inactive [67]. These poised enhancers have been associated primarily with early regulators of cell lineage commitment, and there is very limited evidence that ES cell pluripotency involves protein-DNA interactions at enhancers of terminal differentiation genes [68]. At present, we cannot determine whether the accessibility of PMC CRMs in other lineages reflects the association of these regulatory elements with transcriptional activators or with repressors. In support of the latter, we detected an enrichment of binding sites for HesC in these CRMs. HesC acts as a repressor of skeletogenic genes and presumably interacts with these sites only in non-PMC lineages, where the protein is expressed [69]. We currently favor the hypothesis that CRMs that regulate terminal skeletogenic effector genes are open in non-PMC lineages as a consequence of their association with HesC or other repressors, but this remains to be tested.

Our ATAC-seq analysis of 128-cell stage embryos showed that most of the high-confidence set of overlapping differential peaks, including several experimentally verified PMCs CRMs, were hypersensitive at the 128-cell (late cleavage) stage, several hours prior to the zygotic activation of skeletogenic effector genes. If enhancer priming reflects a pre-activation state, as is widely believed [67, 70], then these findings suggest that pioneer transcription factors (acting singly or in concert with other proteins) interact with PMC CRMs very early in embryogenesis and point to the earliest PMC-specific transcription factors, including Alx1, as candidates. However, as noted above, hyperaccessibility at the 128-cell stage may instead reflect the binding of repressors in non-PMC lineages. Further analysis of purified cell populations will be required to define the temporal and spatial patterns of hyperaccessibility exhibited by PMC CRMs during early embryogenesis.

In previous work we identified 420 genes differentially expressed by PMCs, a gene set that included large numbers of terminal effectors as well as several regulatory genes that had not been previously incorporated into the network [21]. We showed that approximately half of the genes differentially expressed in PMCs were regulated by both Alx and Ets1, although the mechanism of this co-regulation was not explored. In this study, we found a significant enrichment of both Alx1 and Ets1 binding sites in ATAC-seq differential peaks, DNase-seq differential peaks, and the overlapping peak set, suggesting that a large proportion of PMC CRMs receive direct inputs from both Alx1 and Ets1 (or possibly from other homeodomain and ETS family proteins with similar binding sites). Because *Sp-alx1* is positively regulated by Ets1 [17, 35] this suggests that a feedforward mechanism originally proposed by Oliveri and co-workers to account for the regulation of *Sp-msp130, Sp- msp103L*, and *Sp-foxb* [11] may control a large fraction of the effector genes in the PMC GRN. Our studies also point to previously unidentified regulators, as several CRMs active in our reporter gene assay lack consensus binding sites for Alx1, Ets1, or any other transcription factor currently incorporated into the PMC network. In this regard, we also found using de novo motif searching that poly-CT (poly-GA) tracts are significantly enriched in differential peaks compared to peaks that are not differential. The significance of these low-complexity motifs is unknown, but they may be recognized by sequence-specific DNA-binding proteins such as GAGA-binding proteins, chromatin modifiers that bind preferentially to clustered GAGAG elements and are associated with local nucleosome depletion [71, 72].

## Conclusions

We used ATAC-seq to provide a chromatin accessibility map of purified sea urchin PMCs, which are widely used as a developmental model. Cell type-specific hyperaccessibility was used to identify and characterize CRMs that regulate effector genes in the PMC GRN. ATAC-seq and DNase-seq identified 3073 putative CRMs selectively active in these cells, including 161 high-confidence CRMs pinpointed by both strategies. Putative effector gene CRMs were preferentially located near genes expressed selectively by PMCs and a high proportion drove reporter gene expression specifically in PMCs. Consensus binding sites for two key transcription factors, Alx1 and Ets1, were enriched in these CRMs, which also contained disproportionate numbers of repeating CT (or GA) motifs. Surprisingly, CRMs associated with PMC effector genes were hyperaccessible in non-PMC lineages and were open by the 128-cell stage, several hours before gene activation. Our work will enhance the value of the PMC GRN as a general paradigm of developmental GRN architecture and evolution, and will extend its utility as an experimental model for elucidating the genetic regulation of morphogenesis.

## Methods

### *S. purpuratus* embryo culture

Adult *Strongylocentrotus purpuratus* were obtained from Pat Leahy (California Institute of Technology, Pasadena, CA, USA). Gametes were collected from *S. purpuratus* adults by intracoelomic injection of 0.5 M KCl and cultured in artificial seawater at 15°C in a 4-l beaker fitted with a battery-powered stirrer.

### ATAC-seq sample preparation and sequencing

PMCs and a “non-PMC” cell fraction were isolated from early mesenchyme blastula stage embryos at 24 hpf as described previously [21, 73]. As in this previous study, the purity of the PMC fraction was > 90% as determined by the fraction of 6a9-positive cells and the depletion of PMCs from the non-PMC fraction was confirmed by RT-PCR. For generating ATAC-seq libraries, PMCs and the corresponding non-PMC fraction were isolated from three embryo cultures derived from separate matings, which served as biological replicates. In one experiment, ATAC-seq was performed on a single batch of 128-cell *S. purpuratus* embryos.

ATAC-seq was performed following the protocol of Buenrostro and co-workers [41] with minor modifications. Briefly, nuclei were extracted from PMCs and other cells by washing three times with lysis buffer (10 mM Tris-HCl, pH 7.4, 10 mM NaCl, 3 mM MgCl_2_, 0.1% IGEPAL). Nuclei were counted with a hemocytometer. 150,000 nuclei per sample were digested with 2.5 μl transposase (Tn5 transposase from the Nextera kit) at 37°C for 30 min. The digests were purified using the Qiagen minElute PCR purification kit. The purified DNA was amplified using primers against Illumina adaptors for 5 cycles. The number of additional cycles required for optimal amplification of the library was determined using qPCR. The amplified library was purified using the Qiagen minElute PCR purification kit and provided to the USC Epigenome Center for library construction and sequencing. Six libraries (three PMC libraries and three non-PMC cell libraries) were sequenced with an Illumina NextSeq. Approximately 85 million single reads of 76 bp length were obtained per sample.

### DNase-seq sample preparation and sequencing

PMC (−) embryos were produced by treating embryos with U0126, a MEK inhibitor that selectively blocks PMC specification [54, 55]. Embryos were treated with 10 μM U0126 continuously from the 2-cell stage and sibling control embryos were treated with vehicle (DMSO) alone. For DNase-seq analysis, embryos from three separate matings were collected at 28 hpf; these samples served as biological replicates. Several control and UO126-treated embryos from each batch were immunostained with monoclonal antibody 6a9 [64] to confirm that PMC specification was effectively blocked (> 98%) by U0126 treatment. Nuclei from the three batches of U0126-treated and sibling control embryos were isolated as described by Coffman and Yuh [74].

DNase-seq was performed on isolated nuclei as previously described [40]. Briefly, nuclei were digested with 0, 100, 200, 300 and 400 units of DNase I (10 million nuclei per digestion) at 37°C for 3 min in digestion buffer (15 mM Tris-HCl pH 8.0, 15 mM NaCl, 60 mM KCl, 0.5 mM EGTA, 1 mM EDTA, 0.5 mM spermidine). The reaction was stopped by adding stop buffer (50 mM Tris-HCl pH 8.0, 100 mM NaCl, 0.1% SDS, 100 mM EDTA, 10 μg/ml RNase A, 1 mM spermidine, 0.3 mM spermine) and the digested nuclei were treated with Proteinase K overnight at 55°C. Aliquots of digested nuclei were run on a 0.5% agarose gel, and the digest that produced a light smear (typically a digestion with 200–300 units of DNase I) was selected for further processing.

The selected digests were cleaned by phenol-chloroform extraction, layered on a 9% sucrose solution (0.26 M sucrose, 1 M NaCl, 20 mM Tris-HCl pH 8.0, 5 mM EDTA) and ultracentrifuged in a SW41 swinging bucket rotor at 25,000 g for 24 h at 20°C. 600 μL fractions were collected and 10 μL aliquots were run on a 2% agarose gel, stained with SYBR Green I, and imaged with a Typhoon Gel Imager. Fractions containing DNA fragments < 500 bp in size were pooled and mixed with 3X volume of Qiagen QG buffer from the Qiagen MinElute Gel Extraction Kit. 1X volume of isopropanol was added and the samples were purified using Qiagen MinElute columns. Purified DNA was provided to the USC Epigenome Center for library construction (three libraries from PMC-minus embryos and three from sibling control embryos) and Illumina sequencing (HiSeq2000). Approximately 23.5 million single reads of 50 bp length were obtained per sample.

### Analysis of DNase-seq and ATAC-seq data

Raw sequence reads were assessed for quality using FastQC (v0.11.4) [75] and adapter sequences were trimmed using Cutadapt (v1.9) [76]. Reads were mapped to the *S. purpuratus* genome using Bowtie2 (v2.1.0) [77] with default parameters and *S. purpuratus* genome v3.1, obtained from echinobase.org. This is the latest assembly for which a GFF/GTF annotation exists. The v3.1 genome assembly is 826 Mb in size and consists of 32,008 scaffolds with a N50 of 401.6 kb. On average, ~ 80% of the reads in each sample were mapped to the genome assembly by Bowtie2.

Samtools (v1.3) [78] was used to convert the Bowtie2 SAM output format to BAM format. PCR duplications were removed and read counts were equalized using Samtools. Bedtools (v2.19.1) [79] was then used to convert the BAM output into BED format. The BED files were loaded into Fseq (v1.85) [80] to call peaks using parameters -f 0 and -t 2, where -t 2 is a sensitive peak detection threshold. F-Seq has been shown to be a sensitive and accurate peak caller for DNase-seq and ATAC-seq data [81]. The fraction of reads within peaks (the FRiP score) was calculated using Bedtools by extracting and counting all reads within peaks and dividing by the total number of reads mapped. All samples passed a minimum FRiP score threshold of 0.4. Replicate peaks were compared using deepTools [82] and replicates that were found to be highly concordant (Pearson’s correlation coefficient 0.90) were retained. All DNase-seq replicates met this threshold, but one of three ATAC-seq replicates did not meet the threshold and was not considered for further analysis.

Separate reference peak sets (RPSs) were generated for the DNase-seq and ATAC-seq data by first identifying all replicate peaks that overlapped by at least 75% non-reciprocally and then merging all such peaks across samples separately for the DNase-seq or ATAC- seq data using Bedops (v2.4.2) [83]. The 75% overlap criterion was enforced non-reciprocally in order to account for differences in peak sizes across replicates. For example, if a 75% or greater overlap was enforced reciprocally, a peak that was > 25% larger in one replicate or sample would not have been represented in the RPS. Genome coverage of the reference peak sets was determined by first generating a fasta file containing sequences of peaks in the RPS using Bedtools and then counting the number of nucleotides in the fasta file and dividing this by the number of nucleotides in the *S. purpuratus* genome.

Read counts corresponding to peaks in the RPS were generated using HTSeq (v0.6.0) [84] for each replicate. Differential peaks were identified using DESeq2 [85]. Differential peaks in the DNase-seq RPS were identified as peaks that were significantly enriched in the control (whole embryo) replicates compared to the U0126-treated (PMC-deficient) replicates. Peaks were considered significantly enriched if they had nominal *p*-values < 0.1. Differential peaks in the ATAC-seq RPS were identified as peaks that were significantly enriched in the PMC sample compared to the non-PMC sample. Peaks were considered significantly enriched if they had nominal *p*-values < 0.2. A higher p-value threshold was used for ATAC-seq peaks for three reasons: 1) the reduction in the number of replicates (from 3 to 2) compared to the DNase-seq replicates resulted in higher *p*-values assigned to peaks by DESeq2, 2) one well-characterized PMC CRM in our control set (a CRM that regulates the expression of *Sp-tbr*, see Fig. 3d) was detected in the differential peak set at a nominal p-value of 0.18 and would have been missed if a lower threshold were chosen and 3) GFP expression in PMCs was observed when the differential peaks around the *Sp-kirrelL* gene (see Fig. 1b) with nominal *p*-values > 0.1 were cloned along with the peak with nominal *p*-value < 0.1, but not when this peak was cloned alone. Hence, increasing the *p*-value threshold to < 0.2, we were able to capture additional biologically significant peaks. Nominal, and not adjusted, *p*-values were used because multiple hypothesis correction was found to be exceedingly stringent due to the large number of peaks compared.

Overlap between differential peaks identified by DNase-seq and ATAC-seq was determined using Bedops. Differential peaks overlapping non-reciprocally by at least 75% were merged to obtain a set of peaks present in both the ATAC-seq and DNase-seq differential peak sets. Genes within 10 kb of peaks were identified using a custom Python script written by Siddharth Gurdasani. The distribution of peaks with respect to the closest gene and the set of differential peaks within 10 kb of genes differentially expressed by PMCs (PMC DE genes as identified in [21]) were determined. Peak locations with respect to the nearest gene were defined as follows: Upstream (5′): The 3′ end of the peak was within 1–10 kb upstream of the 5′ end of the first exon; Promoter: The 3′ end of the peak was within 1 kb upstream of the 5′ end of the first exon; Within Gene Body: The 5′ end of the peak was within introns or exons; Downstream (3′): The 5′ end of the peak was within 10 kb downstream of, and did not overlap, the 3′ end of the last exon; Distal: No portion of the peak was within 10 kb of a gene. See Additional file 22: Table S16 and Additional file 23: Table S17 for all genes found within 10 kb of differential peaks.

128-cell ATAC-seq sequence reads were processed up to the peak-calling stage as described above.

### CRM validation using GFP reporter plasmids

GFP reporter gene constructs were generated by cloning individual, putative PMC CRMs into the EpGFPII plasmid [56]. Putative PMC CRMs (see Additional file 9: Table S8) along with ~ 200 bp of flanking regions were amplified from *S. purputatus* genomic DNA by PCR and cloned upstream of the basal *Sp-endo16* promoter. In a few cases, adjacent peak regions were also cloned along with the differential peak region. Some constructs also included a promoter peak that was also amplified and cloned upstream of the putative PMC CRM (indicated in Additional file 9: Table S8).

Linearized constructs were injected into *S. purputatus* eggs following established protocols [86]. *S. purpuratus* eggs were fertilized in the presence of 0.1% (wt/vol) para-aminobenzoic acid to prevent hardening of the fertilization envelope. The 20 μl injection solution consisted of 100 ng construct, 500 ng HindIII-digested genomic *S. purputatus* DNA, 0.12 M KCl, 20% glycerol and 0.25% Texas Red dextran. GFP expression was assayed by fluorescence microscopy at the late gastrula stage (48 hpf). Embryos were scored to determine total number of injected embryos (using Texas Red dextran as a marker), the number of embryos showing PMC-specific GFP expression, and the number of embryos with ectopic GFP expression.

### Transcription factor motif detection and analysis

AME (v4.11.2) [87] was used to determine if experimentally verified, sea urchin consensus TF binding sites were enriched in differential peaks compared to non-differential peaks. First, enrichment of the consensus TF binding sites in differential peaks compared to a shuffled control was determined. Any sites not also enriched in non-differential peaks compared to a shuffled control were determined to be enriched in differential peaks compared to non-differential peaks. FIMO (v4.11.2) [58] was used to search peak sets for sea urchin consensus transcription factor binding sites. MEME [60, 61] was used for de novo motif searching.

## Additional files

Additional file 1: **Figure S1.** Sequence analysis pipeline and correlation of DNase-seq and ATAC-seq peaks within replicates. **A)** The bioinformatics pipeline used for DNase- seq and ATAC-seq sequence analysis. **B)** A scatterplot of the read counts of reads aligning to peaks in replicate 1 and 2 of isolated PMCs and other cells of the embryo. Replicates are highly concordant, with an average Pearson’s correlation of 0.915. **C)** A scatterplot of the read counts of reads aligning to peaks in all three replicates of PMC (−) and control embryos. Replicates are highly concordant, with an average Pearson’s correlation of 0.95. (PDF 672 kb)

Additional file 2: **Table S1.** Detailed sequence analysis information for ATAC-seq sequence reads. (DOCX 68 kb)

Additional file 3: **Table S2.** All peaks identified in both PMCs and Other Cells (non-PMCs) at 24 hpf (mesenchyme blastula stage). Column 1: Scaffold: *S. purpuratus* genome version 3.1 Scaffold containing the peak. Column 2: Peak Start: Start coordinate of the peak. Column 3: Peak End: End coordinate of the peak. Column 4: Peak Name. (XLSX 8779 kb)

Additional file 4: **Table S3.** All peaks identified in PMCs at 24 hpf (mesenchyme blastula stage). Column definitions as in Additional file 3: Table S2. (XLSX 6052 kb)

Additional file 5: **Table S4.** All peaks identified in Other Cells (non-PMCs) at 24 hpf (mesenchyme blastula stage). Column definitions as in Additional file 3: Table S2. (XLSX 5816 kb)

Additional file 6: **Table S5.**ATAC-seq peaks significantly differentially enriched (DESSeq2 nominal *p* < 0.2) in isolated PMCs compared to non-PMC cells. Column 1: ATAC-seq Peak Name: Peak number, obtained from ATAC-seq RPS peak names. Column 2: Scaffold: *S. purpuratus* genome version 3.1 scaffold containing the peak. Column 3: Start: Start coordinate of the peak. Column 4: End: End coordinate of the peak. Column 5: baseMean: DESeq2 mean of normalized counts for all samples. Column 6: log2FoldChange: the DESeq log2 fold change of the treatment mean over the control mean. Column 7: lfcSE: log 2 fold change standard error. Column 8: stat: Wald statistic comparing treatment condition to control. Column 9: pvalue: Wald test *p* value. (XLSX 216 kb)

Additional file 7: **Table S19.** ATAC-seq peaks significantly differentially enriched (DESeq2 nominal *p* < 0.2) in Other Cells (non-PMCs) compared to PMCs. Column definitions as in Additional file 6: Table S5. (XLSX 207 kb)

Additional file 8: **Figure S2.** Functional category (GO) enrichment for differential peak sets. A) The functional categorization of genes within 10 kb of ATAC-seq differential peaks. Functional assignments obtained from Echinobase are based on hand annotation (Sea Urchin Genome Sequencing Consortium, 2006) and on primary GO terms derived by blast2go (Tu et al., 2012). Of the 1110 genes within 10 kb of differential peaks, 326 have been assigned to functional categories. Genes assigned to multiple functional classes are counted multiple times. B) The functional categorization of genes within 10 kb of DNase-seq differential peaks. Of the 1216 genes within 10 kb of differerential peaks, 400 have been assigned to functional categories. C) The functional categorization of genes within 10 kb of overlapping, differential peaks. Of the 135 genes within 10 kb of overlapping, differential peaks, 55 have been assigned to functional categories. (PDF 1290 kb)

Additional file 9: **Table S6.** Detailed sequence analysis information for DNase-seq sequence reads. (DOCX 85 kb)

Additional file 10: **Table S7.** Peaks significantly differentially enriched (DESeq2 nominal *p* < 0.1) in control, whole embryos compared to U0126-treated, PMC-minus embryos. Column 1: DNase-seq Peak Name: peak number, obtained from DNase-seq RPS peak names. Column 2-9: as described for Additional file 6: Table S5. (XLSX 227 kb)

Additional file 11: **Table S20.** DNase-seq peaks identified in PMC-minus embryos at 28 hpf (mesenchyme blastula stage). Column 1: Scaffold: *S. purpuratus* genome version 3.1 scaffold containing the peak. Column 2: Peak Start: Start coordinate of the peak. Column 3: Peak end: End coordinate of the peak. (XLSX 2969 kb)

Additional file 12: **Table S21.** DNase-seq peaks significantly differentially enriched (DESeq2 nominal *p* < 0.1) in PMC-minus embryos compared to control embryos. Column definitions as in Additional file 10, Table S7. (XLSX 144 kb)

Additional file 13: **Table S8.** Peaks cloned into EpGFPII and injected into *S. purpuratus* eggs. Column 1: ATAC Peak Name: Peak number obtained from ATAC-seq RPS peak names. These are the peaks cloned into the EpGFPII plasmid. Column 2: Scaffold: *S. purpuratus* genome version 3.1 scaffold containing the peak. Column 3: Start: Start coordinate of the peak. Column 4: End: End coordinate of the peak. Column 5: Name of closest gene. Column 6: PMC DE gene?: "Yes" if the closest gene is a PMC DE gene. Column 7: DNase-seq Diff Peak?: "Yes" if this peak is also differential in the DNase-seq dataset. Column 8: GFP Expression?: "Yes, PMC-specific" if the construct drives PMC-specific GFP expression in 48-hour embryos. Column 9: Region Cloned: The coordinates of the region (containing the peak plus flanking regions) cloned into the EpGFPII plasmid. Column 10: Orientation wrt Closest Gene: "Normal" orientation indicates that the peak was cloned in its normal orientation relative to the coding strand of the closest gene. "Reversed" means that the orientation was reversed relative to the coding strand of the closest gene. Column 11: Notes: Additional information, if applicable, regarding additional peaks cloned into the same construct. (XLSX 52 kb)

Additional file 14: **Table S9.** Enrichment of PMC TF consensus binding sites in differential peaks. Consensus sequences for 14 sea urchin TFs are shown. Binding sites for Ets1 and Alx1, two PMC-enriched TFs, are significantly enriched (*p* < 0.0134) in ATAC-seq, DNase-seq, and overlapping differential peaks. HesC binding sites are significantly enriched (p < 0.0016) in the DNase-seq differential peak set. (DOCX 90 kb)

Additional file 15: **Table S10.** Predicted TF binding sites in experimentally validated PMC CRMs. FIMO (Grant et al., 2011) identified several known sea urchin TF consensus binding sites in PMCs validated by reporter gene assays. (DOCX 51 kb)

Additional file 16: **Table S11.** Coordinates for the set of ATAC-seq differential peaks that overlapped DNase-seq differential peaks by at least 75% merged with the set of DNase-seq peaks that overlapped ATAC-seq differential peaks by at least 75%. Column 1: Scaffold: *S. purpuratus* genome version 3.1 scaffold containing the peak. Column 2: Start: Start coordinate of the peak. Column 3: End: End coordinate of the peak. (XLSX 48 kb)

Additional file 17: **Figure S3.** Sequences enriched in overlapping, differential peaks, as identified by de novo motif discovery. Four motifs were found to be enriched in overlapping, differential peaks compared to non-differential peaks. (PDF 98 kb)

Additional file 18: **Table S12.** Detailed sequence analysis information for 128-cell ATAC-seq sequence reads. (DOCX 49 kb)

Additional file 19: **Table S13.** All ATAC-seq peaks identified at the 128-cell stage. Column 1: Scaffold: *S. purpuratus* genome version 3.1 scaffold containing the peak. Column 2: Peak Start: Start coordinate of the peak. Column 3: Peak End: End coordinate of the peak. Column 4: Peak Name as assigned by F-seq. Column 5: Peak Score as assigned by F-seq. (XLSX 16076 kb)

Additional file 20: **Table S14.** Coordinates of all differential peaks found using ATAC-seq or DNase-seq that are also open at the 128-cell stage. (XLSX 75 kb)

Additional file 21: **Table S15.** Coordinates of overlapping, differential peaks that are also open at the 128-cell stage. (XLSX 44 kb)

Additional file 22: **Table S16.** Transcripts within 10 kb of ATAC-seq differential peaks. Column 1: Peak name: Peak number, obtained from ATAC-seq RPS peak names. Column 2: Scaffold: *S. purpuratus* genome version 3.1 scaffold containing the peak. Column 3: Start: Start coordinate of the peak. Column 4: End: End coordinate of the peak. Column 5: Transcript: Transcript within 10 kb of the peak. Column 6: Location of peak wrt transcript: Location definitions as described in the Methods. Column 7: Distance in bp: Distance of the peak from the transcript in base pairs. Column 8: Name of PMC DE gene: Name of the PMC DE gene withing 10 kb of the peak, if applicable. (XLSX 157 kb)

Additional file 23: **Table S17.** Transcripts within 10 kb of DNase-seq differential peaks. Column 1: Peak name: Peak number, obtained from DNase-seq RPS peak names. Columns 2-8: as described for Additional file 22: Table S16. (XLSX 178 kb)

## Abbreviations

ATAC-seq: Assay for transposase-accessible chromatin using sequencing
CRM: Cis-regulatory module
DE: Differentially expressed
DNase-seq: DNase I hypersensitive site sequencing
GRN: Gene regulatory network
PMC: Primary mesenchyme cell
