# Supplementary material for: Global analysis of primary mesenchyme cell cis-regulatory modules by chromatin accessibility profiling
Source: BMC Genomics. 2018 Mar 20;19:206. doi: 10.1186/s12864-018-4542-z (PMC5859501; doi:10.1186/s12864-018-4542-z)
Supplement: Supplementary file 1 — Figure S1. Sequence analysis pipeline and correlation of DNase-seq and ATAC-seq peaks within replicates. A) The bioinformatics pipeline used for DNase- seq and ATAC-seq sequence analysis. B) A scatterplot of the read counts of reads aligning to peaks in replicate 1 and 2 of isolated PMCs and other cells of the embryo. Replicates are highly concordant, with an average Pearson’s correlation of 0.915. C) A scatterplot of the read counts of reads aligning to peaks in all three replicates of PMC (−) and control embryos. Replicates are highly concordant, with an average Pearson’s correlation of 0.95. (PDF 672 kb) [file 12864_2018_4542_MOESM1_ESM.pdf]

A

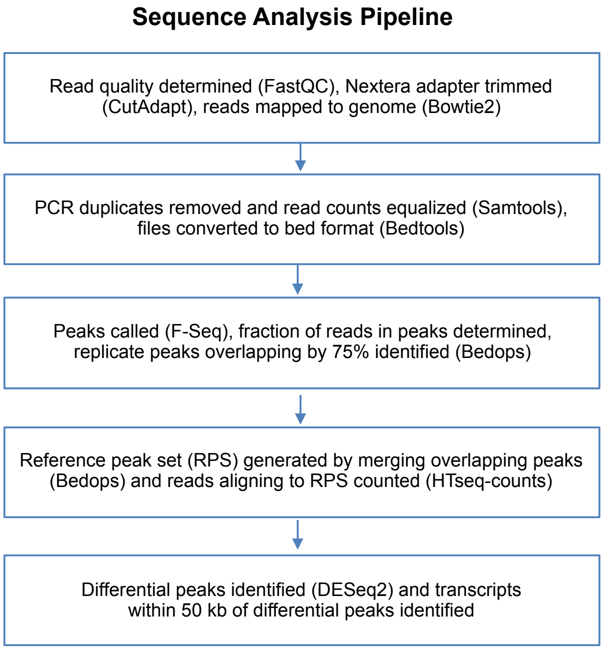
Sequence Analysis Pipeline
Read quality determined (FastQC), Nextera adapter trimmed (CutAdapt), reads mapped to genome (Bowtie2)
PCR duplicates removed and read counts equalized (Samtools), files converted to bed format (Bedtools)
Peaks called (F-Seq), fraction of reads in peaks determined, replicate peaks overlapping by 75% identified (Bedops)
Reference peak set (RPS) generated by merging overlapping peaks (Bedops) and reads aligning to RPS counted (HTseq-counts)
Differential peaks identified (DESeq2) and transcripts within 50 kb of differential peaks identified

B

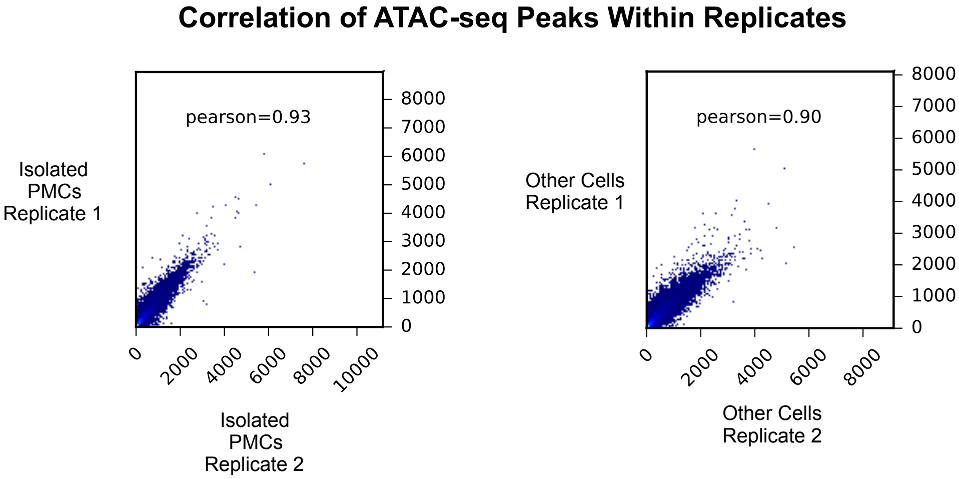
Correlation of ATAC-seq Peaks Within Replicates
pearson=0.93
Isolated PMCs Replicate 1
Isolated PMCs Replicate 2
pearson=0.90
Other Cells Replicate 1
Other Cells Replicate 2

C

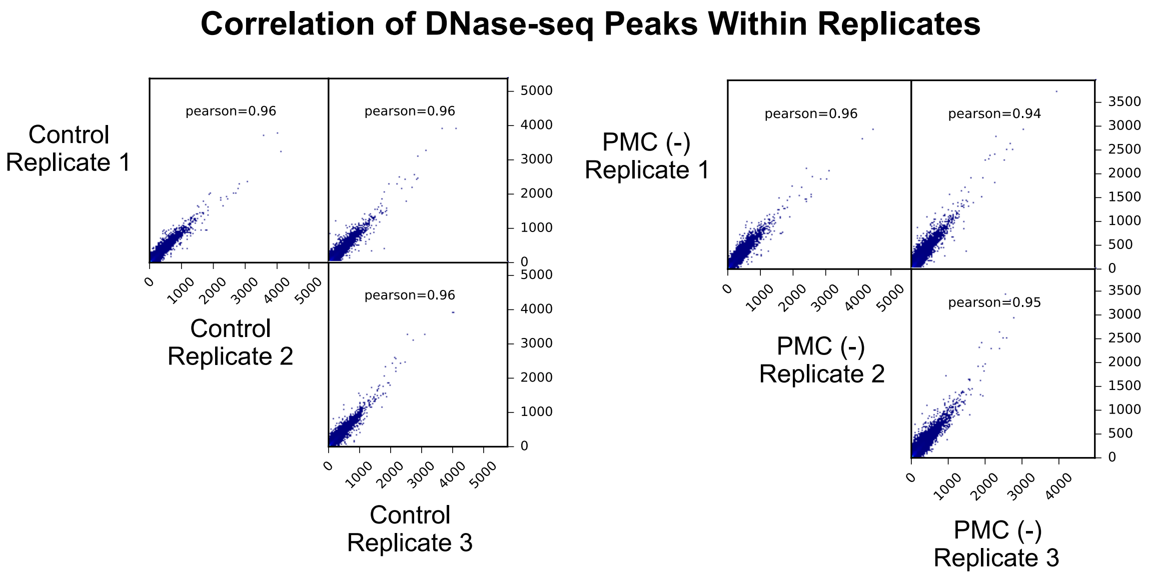
Correlation of DNase-seq Peaks Within Replicates
Control Replicate 1
pearson=0.96
pearson=0.96
pearson=0.96
Control Replicate 2
Control Replicate 3
PMC (-) Replicate 1
pearson=0.96
pearson=0.94
pearson=0.95
PMC (-) Replicate 2
PMC (-) Replicate 3
