# Supplementary material for: Global analysis of primary mesenchyme cell cis-regulatory modules by chromatin accessibility profiling
Source: BMC Genomics. 2018 Mar 20;19:206. doi: 10.1186/s12864-018-4542-z (PMC5859501; doi:10.1186/s12864-018-4542-z)
Supplement: Supplementary file 2 — Table S1. Detailed sequence analysis information for ATAC-seq sequence reads. (DOCX 68 kb) [file 12864_2018_4542_MOESM2_ESM.docx]

**Supplementary Table 4: Sequencing and Peak information for ATAC-seq samples**

| Sample | Number of reads sequenced | Number of mapped reads | Number of reads post duplicate removal and equalization | Number of peaks (f-seq*) | Avg. Peak size (bp) | FRiP Score** |
| --- | --- | --- | --- | --- | --- | --- |
| Isolated PMCs replicate 1 | 75,312,725 | 57,443,004  (76.3%) | 42,992,104 | 361,901 | 502 | 0.64 |
| Isolated PMCs replicate 2 | 79,841,016 | 58,259,643  (72.97%) | 42,999,562 | 379,901 | 500 | 0.62 |
| Other cells replicate 1 | 95,075,858 | 75,936,782  (79.9%) | 42,988,015 | 335,047 | 513 | 0.66 |
| Other cells replicate 2 | 105,842,445 | 85,653,116  (80.9%) | 42,999,562 | 391,604 | 504 | 0.62 |

*F-seq parameters used: -f 0 and –t 2

**FRiP score is calculated by dividing the number of aligned reads overlapping peaks with the total number of reads mapped.
