# Supplementary material for: Global analysis of primary mesenchyme cell cis-regulatory modules by chromatin accessibility profiling
Source: BMC Genomics. 2018 Mar 20;19:206. doi: 10.1186/s12864-018-4542-z (PMC5859501; doi:10.1186/s12864-018-4542-z)
Supplement: Supplementary file 8 — Figure S2. Functional category (GO) enrichment for differential peak sets. A) The functional categorization of genes within 10 kb of ATAC-seq differential peaks. Functional assignments obtained from Echinobase are based on hand annotation (Sea Urchin Genome Sequencing Consortium, 2006) and on primary GO terms derived by blast2go (Tu et al., 2012). Of the 1110 genes within 10 kb of differential peaks, 326 have been assigned to functional categories. Genes assigned to multiple functional classes are counted multiple times. B) The functional categorization of genes within 10 kb of DNase-seq differential peaks. Of the 1216 genes within 10 kb of differerential peaks, 400 have been assigned to functional categories. C) The functional categorization of genes within 10 kb of overlapping, differential peaks. Of the 135 genes within 10 kb of overlapping, differential peaks, 55 have been assigned to functional categories. (PDF 1290 kb) [file 12864_2018_4542_MOESM8_ESM.pdf]

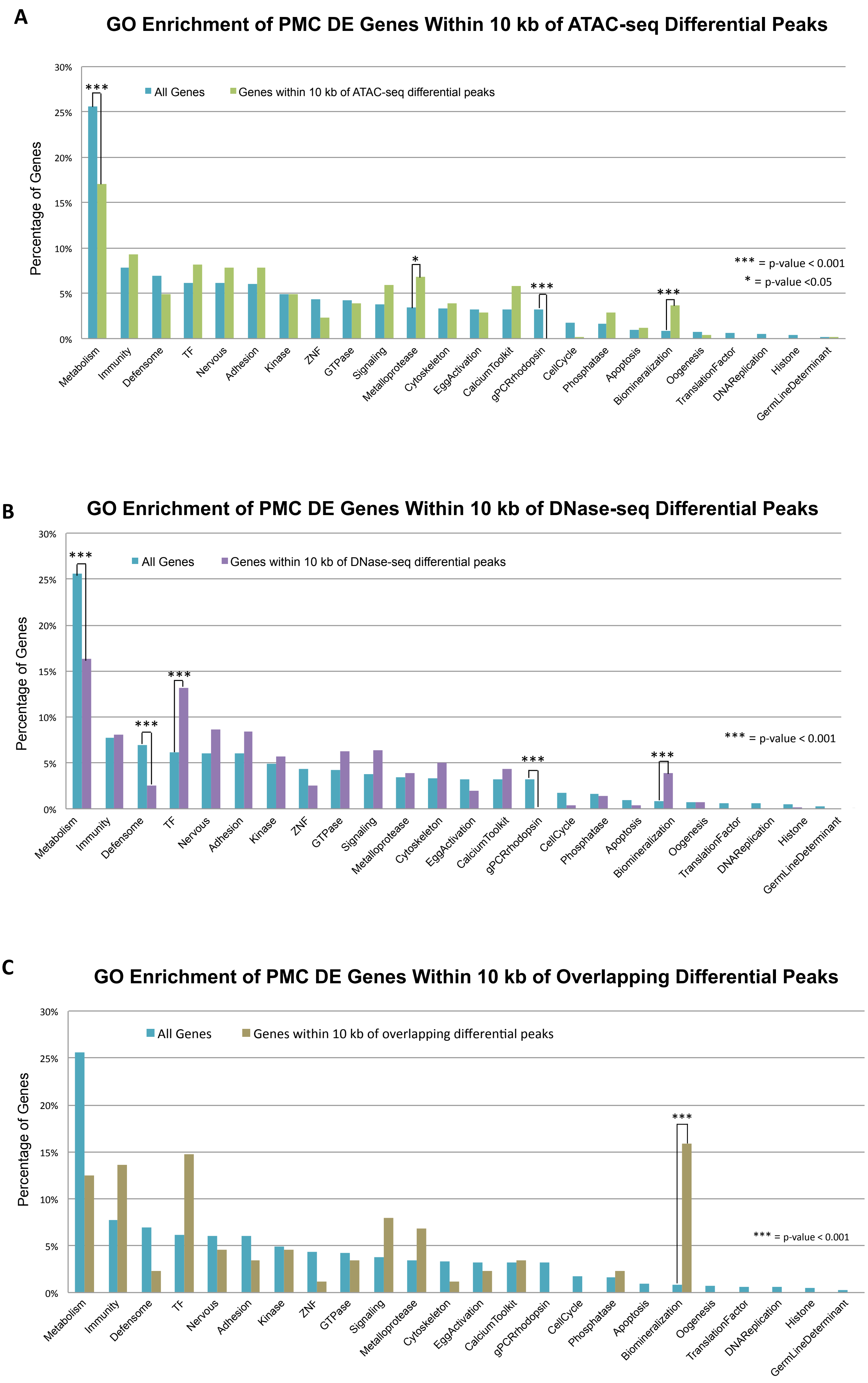
A
GO Enrichment of PMC DE Genes Within 10 kb of ATAC-seq Differential Peaks
All Genes
Genes within 10 kb of ATAC-seq differential peaks
Percentage of Genes
30%
25%
20%
15%
10%
5%
0%
*** = p-value < 0.001
* = p-value <0.05
Metabolism
Immunity
Defensome
TF
Nervous
Adhesion
Kinase
ZNF
GTPase
Signaling
Metalloprotease
Cytoskeleton
EggActivation
CalciumToolkit
gPCRrhodopsin
CellCycle
Phosphatase
Apoptosis
Biomineralization
Oogenesis
TranslationFactor
DNAReplication
Histone
GermLineDeterminant
B
GO Enrichment of PMC DE Genes Within 10 kb of DNase-seq Differential Peaks
All Genes
Genes within 10 kb of DNase-seq differential peaks
Percentage of Genes
30%
25%
20%
15%
10%
5%
0%
*** = p-value < 0.001
Metabolism
Immunity
Defensome
TF
Nervous
Adhesion
Kinase
ZNF
GTPase
Signaling
Metalloprotease
Cytoskeleton
EggActivation
CalciumToolkit
gPCRrhodopsin
CellCycle
Phosphatase
Apoptosis
Biomineralization
Oogenesis
TranslationFactor
DNAReplication
Histone
GermLineDeterminant
C
GO Enrichment of PMC DE Genes Within 10 kb of Overlapping Differential Peaks
All Genes
Genes within 10 kb of overlapping differential peaks
Percentage of Genes
30%
25%
20%
15%
10%
5%
0%
*** = p-value < 0.001
Metabolism
Immunity
Defensome
TF
Nervous
Adhesion
Kinase
ZNF
GTPase
Signaling
Metalloprotease
Cytoskeleton
EggActivation
CalciumToolkit
gPCRrhodopsin
CellCycle
Phosphatase
Apoptosis
Biomineralization
Oogenesis
TranslationFactor
DNAReplication
Histone
GermLineDeterminant
