# Supplementary material for: Global analysis of primary mesenchyme cell cis-regulatory modules by chromatin accessibility profiling
Source: BMC Genomics. 2018 Mar 20;19:206. doi: 10.1186/s12864-018-4542-z (PMC5859501; doi:10.1186/s12864-018-4542-z)
Supplement: Supplementary file 9 — Table S6. Detailed sequence analysis information for DNase-seq sequence reads. (DOCX 85 kb) [file 12864_2018_4542_MOESM9_ESM.docx]

**Supplementary Table 3: Sequencing and Peak information for DNase-seq samples**

| Sample | Number of reads sequenced | Number of mapped reads | Number of reads post duplicate removal and equalization | Number of peaks (f-seq*) | Avg. Peak size (bp) | FRiP Score** |
| --- | --- | --- | --- | --- | --- | --- |
| Control whole 28 hpf embryos replicate 1 | 26,403,533 | 21,043,220 (79.70%) | 14,677,169 | 244,614 | 443 | 0.55 |
| Control whole 28 hpf embryos replicate 2 | 23,619,516 | 19,044,489 (80.63%) | 14,676,742 | 247,373 | 454 | 0.55 |
| Control whole 28 hpf embryos replicate 3 | 23,895,538 | 19,219,813 (80.43%) | 14,680,727 | 259,415 | 429 | 0.54 |
| PMC-minus 28hpf embryos replicate 1 | 20,303,626 | 16,262,383 (80.10%) | 14,678,403 | 241,839 | 447 | 0.56 |
| PMC-minus 28hpf embryos replicate 2 | 24,287,454 | 19,288,663 (79.42%) | 14,678,452 | 258,675 | 459 | 0.55 |
| PMC-minus 28hpf embryos replicate 3 | 22,812,500 | 18,295,936 (80.20%) | 14,679,972 | 284,124 | 418 | 0.52 |

*F-seq parameters used: -f 0 and –t 2

**FRiP score is calculated by dividing the number of aligned reads overlapping peaks with the total number of reads mapped.
