# Supplementary material for: Global analysis of primary mesenchyme cell cis-regulatory modules by chromatin accessibility profiling
Source: BMC Genomics. 2018 Mar 20;19:206. doi: 10.1186/s12864-018-4542-z (PMC5859501; doi:10.1186/s12864-018-4542-z)
Supplement: Supplementary file 14 — Table S9. Enrichment of PMC TF consensus binding sites in differential peaks. Consensus sequences for 14 sea urchin TFs are shown. Binding sites for Ets1 and Alx1, two PMC-enriched TFs, are significantly enriched (p < 0.0134) in ATAC-seq, DNase-seq, and overlapping differential peaks. HesC binding sites are significantly enriched (p < 0.0016) in the DNase-seq differential peak set. (DOCX 90 kb) [file 12864_2018_4542_MOESM14_ESM.docx]

**Enrichment of PMC TF Consensus Binding Sites in Differential Peaks.**

| **Transcription**  **Factor** | **Consensus**  **Sequence** | **Citation** | **Enrichment** |
| --- | --- | --- | --- |
| Ets1 | (C/A)GGAA or  A(C/A)C(C/A)GGAA(C/G)TA | Damle and Davidson, 2011; Consales and Arnone, 2001 | DNase-seq, ATAC-seq and overlapping peaks |
| Alx1 | TAATNNNATTA | Damle and Davidson, 2011 | DNase-seq, ATAC-seq and overlapping peaks |
| Blimp1 | G(A/G)AA(C/G)(G/T)GAAA; G(A/G)AA(C/G)AAAN | Yuh et. al., 2004 | None |
| Tbr | AGGTGTGA; AGGTGACA | Jarvela et. al., 2014 | None |
| Tcf1 | TTCAAAGG | Yuh et. al., 2004 | None |
| Gata | (C/T)GATA(A/G) | Lowry and Atchley, 2000 | None |
| Otx | TAATC(C/T) | Yuh et. al., 2004 | None |
| HesC | CACGTG or  CACGCG | Ochiai et. al., 2008;  Smith and Davidson, 2008 | DNase-seq peaks |
| bZIP | GCCGATTCAT | Range et. al., 2007 | None |
| Sox | AACAAT | Range et. al., 2007 | None |
| Myb | YAA(CG/TG) | Range et. al., 2007 | None |
| Ot1 | ATGCTAAA | Range et. al., 2007 | None |
| Gcm | ATRCGGGY | Calestani and Rogers, 2010 | None |
| CBF | CCAATT | Dayal et. al., 2004 | None |
