# Supplementary material for: Global analysis of primary mesenchyme cell cis-regulatory modules by chromatin accessibility profiling
Source: BMC Genomics. 2018 Mar 20;19:206. doi: 10.1186/s12864-018-4542-z (PMC5859501; doi:10.1186/s12864-018-4542-z)
Supplement: Supplementary file 15 — Table S10. Predicted TF binding sites in experimentally validated PMC CRMs. FIMO (Grant et al., 2011) identified several known sea urchin TF consensus binding sites in PMCs validated by reporter gene assays. (DOCX 51 kb) [file 12864_2018_4542_MOESM15_ESM.docx]

| **Validated PMC**  **CRM** | **Predicted TF**  **Binding Sites** |
| --- | --- |
| *Sp-kirrelL* (WHL22.699052) | Sox, Tbr, Gcm, bZIP, Otx, Myb, Ets1 |
| *Sp-mitf* (WHL22.677144) | Sox |
| *Sp-msp130r2* (WHL22.451280) | HesC, Blimp1, Gata, Sox |
| *Sp-sh2d5* (WHL22.637506) | Ets1, Gata, Gcm |
| *SPU_023052* (WHL22.364101) | None |
| Novel PMC DE Gene (WHL22.691495) | Myb |
| *Sp-hypp_2386* (WHL22.239326) | Blimp, Alx1 |
| *Sp-c-lectin/PMC1* (WHL22.411805) | Otx, Ets1 |
| Intergenic | None |

**Predicted TF Binding Sites in Validated PMC CRMs**
