# Supplementary figures and images for: Global analysis of primary mesenchyme cell cis-regulatory modules by chromatin accessibility profiling
Source: BMC Genomics. 2018 Mar 20;19:206. doi: 10.1186/s12864-018-4542-z (PMC5859501; doi:10.1186/s12864-018-4542-z)

| Motif Logo                                                                         | No. of Sites | MEME E-value |
|------------------------------------------------------------------------------------|--------------|--------------|
| 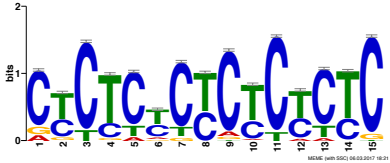  | 119          | 2.2E-176     |
| 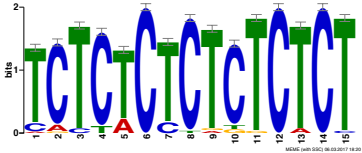  | 39           | 2.6E-86      |
| 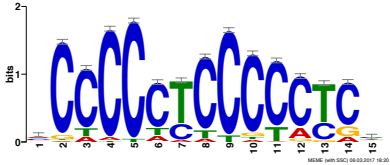  | 61           | 5.9E-66      |
| 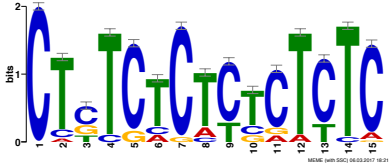 | 40           | 5.9E-29      |

Supplement: Supplementary file 17 — Figure S3. Sequences enriched in overlapping, differential peaks, as identified by de novo motif discovery. Four motifs were found to be enriched in overlapping, differential peaks compared to non-differential peaks. (PDF 98 kb) [file 12864_2018_4542_MOESM17_ESM.pdf]
