# Supplementary material for: Global analysis of primary mesenchyme cell cis-regulatory modules by chromatin accessibility profiling
Source: BMC Genomics. 2018 Mar 20;19:206. doi: 10.1186/s12864-018-4542-z (PMC5859501; doi:10.1186/s12864-018-4542-z)
Supplement: Supplementary file 18 — Table S12. Detailed sequence analysis information for 128-cell ATAC-seq sequence reads. (DOCX 49 kb) [file 12864_2018_4542_MOESM18_ESM.docx]

**Supplementary Table: Sequencing and Peak information for 128-cell ATAC-seq Sample**

| Sample | Number of reads sequenced | Number of mapped reads | Number of reads post duplicate removal | Number of peaks (f-seq*) | Avg. Peak size (bp) | FRiP Score** |
| --- | --- | --- | --- | --- | --- | --- |
| Control whole 28 hpf embryos replicate 1 | 97,645,821 | 60,118,344 (61.57%) | 41,651,211 | 360,959 | 498 |  |

*F-seq parameters used: -f 0 and –t 2

**FRiP score is calculated by dividing the number of aligned reads overlapping peaks with the total number of reads mapped.
